# Do Age and Linguistic Status Alter the Effect of Sound Source Diffuseness on Speech Recognition in Noise?

**DOI:** 10.3389/fpsyg.2022.838576

**Published:** 2022-03-15

**Authors:** Meital Avivi-Reich, Rupinder Kaur Sran, Bruce A. Schneider

**Affiliations:** ^1^Department of Communication Arts, Sciences and Disorders, Brooklyn College, City University of New York, Brooklyn, NY, United States; ^2^Human Communication Lab, Department of Psychology, University of Toronto Mississauga, Toronto, ON, Canada; ^3^Department of Speech-Language Pathology, University of Toronto, Toronto, ON, Canada

**Keywords:** masking, aging, bilingualism, speech perception, diffuseness, amplification

## Abstract

One aspect of auditory scenes that has received very little attention is the level of diffuseness of sound sources. This aspect has increasing importance due to growing use of amplification systems. When an auditory stimulus is amplified and presented over multiple, spatially-separated loudspeakers, the signal’s timbre is altered due to comb filtering. In a previous study we examined how increasing the diffuseness of the sound sources might affect listeners’ ability to recognize speech presented in different types of background noise. Listeners performed similarly when both the target and the masker were presented via a similar number of loudspeakers. However, performance improved when the target was presented using a single speaker (compact) and the masker from three spatially separate speakers (diffuse) but worsened when the target was diffuse, and the masker was compact. In the current study, we extended our research to examine whether the effects of timbre changes with age and linguistic experience. Twenty-four older adults whose first language was English (Old-EFLs) and 24 younger adults whose second language was English (Young-ESLs) were asked to repeat non-sense sentences masked by either Noise, Babble, or Speech and their results were compared with those of the Young-EFLs previously tested. Participants were divided into two experimental groups: (1) A Compact-Target group where the target sentences were presented over a single loudspeaker, while the masker was either presented over three loudspeakers or over a single loudspeaker; (2) A Diffuse-Target group, where the target sentences were diffuse while the masker was either compact or diffuse. The results indicate that the Target Timbre has a negligible effect on thresholds when the timbre of the target matches the timbre of the masker in all three groups. When there is a timbre contrast between target and masker, thresholds are significantly lower when the target is compact than when it is diffuse for all three listening groups in a Noise background. However, while this difference is maintained for the Young and Old-EFLs when the masker is Babble or Speech, speech reception thresholds in the Young-ESL group tend to be equivalent for all four combinations of target and masker timbre.

## Introduction

Daily communication takes place in a variety of complex auditory settings that often contain several sound sources, some natural and some amplified. These competing sound sources make it difficult to extract a speech target masked by one or more competing sounds. A number of studies have examined different aspects of auditory scenes to be able to better understand how they may affect speech perception and comprehension. For example, researchers have examined how listening to and processing a speech target is affected by the number of auditory sound sources (e.g., Rosen et al., 2013), their intensity (e.g., Dos Santos Sequeira et al., 2010), spectral composition (e.g., Li and Fu, 2010; Roberts and Summers, 2020), and spatial location (e.g., Ezzatian et al., 2010; Avivi-Reich et al., 2014; Gygi and Shafiro, 2014; Bednar and Lalor, 2020). These studies contributed to our understanding of how the auditory scene and the acoustic input may affect the ways in which listeners detect, process, and encode acoustic signals and verbal information. One aspect of auditory scenes that has received very little attention is how the level of diffuseness of the sound sources affect speech recognition. This topic is becoming increasingly important given the increasing use of surround sound systems in our everyday lives.

Often, when amplification is used, a natural sound source (typically with a compact and defined location) is amplified and presented over more than a single loudspeaker. When an auditory stimulus (e.g., a human voice) is amplified and presented over multiple, spatial-separated loudspeakers, the signal’s timbre is altered due to comb filtering, and the sound source is perceived to be more diffuse and with a broader auditory source width (Avivi-Reich et al., 2020). With the growing use of electric amplification and surround-sound systems, it would be useful to determine how the relative diffuseness and compactness of different sound sources affect speech recognition.

In a previous study (Avivi-Reich et al., 2020) we systematically examined how manipulating the diffuseness of the sound sources might affect the ability of young people with normal hearing to correctly identify target speech presented in different types of background noise. Twenty-four young adults were asked to repeat nonsense sentences that were presented in either Noise, Babble, or competing Speech. Participants were divided into two groups: (1) A Compact-Target group where the target sentences were presented over a single loudspeaker (compact target), while the masker was either presented over three spatially separated loudspeakers (diffuse masker) or over a single loudspeaker (compact); (2) A Diffuse-Target group, where the target sentences were diffuse while the masker was either compact or diffuse. The results of this study showed no significant Timbre effect in the absence of a timbre contrast (compact vs. diffuse) between target and masker. However, when there was a timbre contrast, the signal-to-noise ratios (SNRs) needed for 50% correct recognition of the target speech were higher when the masker was compact, and the target was diffuse, and lower when the target was compact, and the masker was diffuse. These results were consistent with the expected effects from comb filtering (for additional information and illustrations see Avivi-Reich et al., 2020), and also could reflect a tendency for attention to be drawn toward compact sound sources that may be perceived as closer in order to avoid dangerous situations or objects even without seeing them (Scharf, 1998; Farnè and Làdavas, 2002; Canzoneri et al., 2012). In vision, the tendency of closer items to have higher ecological salience is referred to as the behavior urgency hypothesis (Franconeri and Simons, 2003). These findings emphasize the importance of considering the level of diffuseness when designing and using amplification systems, especially when using amplification in order to enhance speech perception.

Speech perception in noise (SPIN) can be a demanding task both at peripheral and more central processing levels. Any competing sources in the auditory scene that temporally and spectrally overlaps the target speech signal creates overlapping excitation patterns in the cochlea and in the auditory nerve. This overlap might interfere with the perception and processing of the target at the auditory periphery, which often is referred to as energetic masking or peripheral masking (Durlach et al., 2003). In addition, when the masker contains meaningful speech, it is likely to initiate lexical processing of the masker, potentially allowing the content of irrelevant streams to intrude into working memory and interfere with the processing of the target message. This type of interference often is referred to as informational masking (Freyman et al., 1999; Durlach et al., 2003; Schneider et al., 2007, 2010; Kidd et al., 2008). While energetic masking seems to affect the early stages of sound perception and processing, informational masking is likely to affect later processes (Arbogast et al., 2002; Freyman et al., 2004; Ihlefeld and Shinn-Cunningham, 2008; Szalárdy et al., 2019).

Listeners can alleviate the effects of informational masking if they are able to segregate the different incoming auditory streams so that attention can be focused on processing the target stream. The ability to successfully segregate the streams largely depends on the perceptual similarities and dissimilarities between the target signal and other competing sound sources. Any differences among the sound sources could assist the listener in perceptually segregating the target stream from the competing sound sources, thereby providing a release from masking (Bregman, 1990). A large number of acoustic cues that could assist auditory stream segregation have been previously investigated in order to assess their potential to release the target signal from masking (e.g., Brungart et al., 2001; Humes et al., 2006; Vongpaisal and Pichora-Fuller, 2007). In the current study, we intend to continue investigating the possible role that timbre differences might play in auditory stream segregation (Bregman, 1990). This cue has received limited attention in the literature (see, for example, Freyman et al., 1999), and as far as we know our previous study was the first to systematically investigate its effect on speech recognition.

The current study aims to extend the previous study (Avivi-Reich et al., 2020) to populations other than young native-English listeners (Young-EFLs) to those who are known to experience greater difficulties when listening in complex auditory environment and may be affected differently by the diffuseness level of the different sound sources. Two such groups, whose ability to perceive speech in noise have been extensively studied, are older adult listeners for whom English is a first language (Old-EFLs) as well as young adults for whom English is their second language (Young-ESLs). These two groups have been found to require more preferable listening conditions in order to achieve correct speech perception compared with young-EFL listeners (e.g., Rogers et al., 2006; Avivi-Reich et al., 2014, 2015; Francis et al., 2018). However, the reasons for their poorer SPIN are likely to be quite different and therefore the effect of sound source diffuseness on their SPIN may differ as well.

### Aging and Speech Perception

Older adults often experience greater difficulties perceiving speech in noisy environments, even those who are considered to have normal hearing (Helfer and Freyman, 2008; Stevenson et al., 2015). Interestingly, not all types of maskers have a similar effect on younger and older listeners. One type of masker that seems particularly detrimental to older adults is competing speech (Tun and Wingfield, 1999; Helfer and Freyman, 2008; Goossens et al., 2017). It has also been suggested that older adults with normal hearing for their age benefit less than younger adults when the target voice and competing sound sources occupy different positions in space (Murphy et al., 2006; Marrone et al., 2008; Avivi-Reich et al., 2014), and when there are fluctuations in the masker signal (Stuart and Phillips, 1996; Dubno et al., 2003; Gifford et al., 2007). In addition, evidence suggests that older adults require a greater amount of time to establish stream segregation when listening in an environment that contains more than a single sound source compared to younger adults (Ben-David et al., 2012; Getzmann and Näätänen, 2015). Considering these age-related findings, it is important to examine if and how older adults’ speech perception may be affected by changes in the diffuseness level of the sound sources in a noisy environment.

There are several possible reasons why older adults may be less able to use differences in diffuseness between target speech and competing sound sources to unmask the target speech. For example, when the masker is diffuse and the target is compact, older adults might not be able to fully use the troughs in the masker spectrum created by the comb filtering effect to improve speech perception (see Avivi-Reich et al., 2020 for more information regarding the effect of comb filtering under the different testing conditions). Other possible reasons may be related to age- related changes in the ability of listeners to form an auditory image of a diffuse vs. a compact sound, their ability to establish stream segregation between sound sources that are either presented over multiple loudspeakers or a single one, and/or their ability to focus their attention on the target stream.

### Second Language and Speech Perception

When listening to a second language, listeners have lower performance than when listening to their first language on a number of speech perception measures (e.g., Ezzatian et al., 2010; Francis et al., 2018; Peng and Wang, 2019). This could be due, in part, to incomplete acquisition of the acoustic–phonetic characteristics in the second language. This incomplete knowledge might result in a reduced phoneme recognition in one’s second or third language (Kroll and Steward, 1994). In addition, non-native listeners’ second language semantic and linguistic processes may not be completely differentiated from their first language processes (FitzPatrick and Indefrey, 2009). This overlap between the two linguistic systems could result in greater competition as both systems are activated when listening. Hence, the degree and extent to which second language listeners might engage knowledge-driven processes (e.g., vocabulary and linguistic knowledge) to facilitate speech perception could differ from the pattern of engagement in the listeners’ first language (Meador et al., 2000). In addition, this greater competition may require greater investment of attentional resources, leaving fewer resources available to attend to fine acoustic changes, such as those created by the presentation of a sound source over several loudspeakers rather than a single one.

## Materials and Methods

### Participants

Twenty-four older listeners for whom English is their first language (Old-EFLs) and 24 younger listeners for whom English is their second language (Young-ESLs) participated in this study. Each group of participants was divided into two experimental groups. Twelve of the Old-EFLs (mean age: 73.08 years; *SD*: 4.60) and 12 of the Young-ESLs (mean age: 21.19 years; *SD*: 1.57) were tested using a compact target speech source (T_C_); and of the other 12 Old-EFLs (mean age: 72.75 years; *SD*: 4.18) and 12 Young-ESLs (mean age: 21.02 years; *SD*: 1.95) were tested using a diffused target speech source (T_d_). Listeners in the Old-EFL group were all born and raised in a country in which the primary language was English and were not fluent in any other language at the time of participation. Listeners in the Young-ESL were born and raised in a language other than English and did not attend an English or an American school before relocating to an English-speaking country at the age of 11 years old or later. The Young-ESL listeners were from a diverse linguistic background (1 Hindi, 1 Philipino, 1 Spanish, 1 Sinhalese, 1 Macedonian, 1 Indonesian, 1 Korean, 1 Russian, 4 Arabic, 2 Portuguese, 1 Malayalam, 1 Cantonese, 8 Mandarin). Their average age at the time of the relocation was 16.21 years (*SD* = 3.15). Participants were recruited from the University of Toronto Mississauga’s Human Communication Lab database system. The database consists of younger adults who are students at the University of Toronto Mississauga and older adults who were individuals living independently in the community from the surrounding area (Mississauga, ON), who provided their own means of transportation to the laboratory. All participants completed a questionnaire regarding their general health, hearing, vision, and cognitive status. Only participants who reported that they were in good health and had no history of serious pathology were included. Participants had normal hearing for their age and no history of hearing disorders or previous use of hearing aids. The study reported here was approved by the Ethics Review Board of the University of Toronto.

### Materials, Apparatus, and Procedure

All participants completed an Audiometric hearing test, the Nelson-Denny reading comprehension test (Brown et al., 1981), and the Mill Hill vocabulary test (Raven, 1965) during the first experimental session. The speech recognition task was administered during a second experimental session. Each of the two sessions was typically 1–1.5 h in duration. All participants provided their written informed consent to participate and were compensated monetarily for their participation.

#### Hearing Measures

##### Audiometric Testing

**FIGURE 1:**
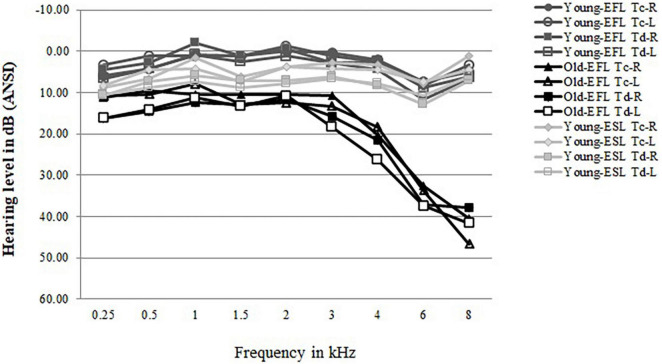
Average audiograms for the two Old-EFL groups (T_C_ vs. T_d_) and the two Young-ESL groups (T_d_ vs. T_C_) as well as for the equivalent two Young-EFL groups from Avivi-Reich et al. (2020). Left and right ears are plotted separately.

Pure-tone air-conduction thresholds were measured at nine frequencies (0.25–8 kHz) for both ears using an Interacoustics Model AC5 audiometer (Interacoustic, Assens, Denmark). All Young-ESL participants were required to have a pure tone threshold of 15 dB HL or lower from 0.25 to 8 kHz but were allowed to have one 20 dB HL threshold in one tested frequency in each ear. All Old-EFL participants were required to have a pure tone threshold of 25 dB HL or lower from 0.25 to 3 kHz. Older adults with hearing thresholds in the range described are usually considered to have normal hearing for their age (ISO 7029-2000). In addition, participants who demonstrated unbalanced hearing (more than 15 dB difference between ears at any tested frequency between 0.25 to 8 kHz) were excluded from participation. Figure 1 plots the average audiometric thresholds for the left and right ears of the Old-EFLs and Young-ESLs in the present study along with the Young-EFLs in Avivi-Reich et al. (2020), separately for the two target groups (T_C_ vs. T_d_).

#### Language Proficiency Measures

##### Vocabulary Knowledge

Participants were asked to complete the Mill Hill vocabulary test (Raven, 1965), which is a 20-item synonym test. In this task, participants were required to choose the closest synonym of each test item from a list of six alternatives. No time restraints were applied.

##### Reading Comprehension Skill

The Nelson-Denny test (Brown et al., 1981) was used to assess the reading comprehension skills of each participant. In this test, the participants had to read through eight independent passages and answer multiple-choice questions based on the content of the passages. This test includes a total of 36 questions and was limited to 20 min. Participants were instructed to answer as many questions as possible within the allotted time.

#### Semantically Anomalous Sentences-Recognition Task

The procedure for the sentence-recognition task was replicated from Avivi-Reich et al. (2020). In the experimental recognition task, listeners sat in a chair placed in the center of an Industrial Acoustic Company (IAC) sound-attenuated chamber. The internal dimensions of this chamber were 283 cm in length, 274 cm in width, and 197 cm in height. As described in Avivi-Reich et al. (2020), two loudspeakers were placed at 45° to the left and right of the listener, with a third placed directly in front of the listener. The distance between the center of the listener’s head and each of the three loudspeakers was about 170 cm. The height of each loudspeaker was adjusted to match the ear level of a seated listener with an average body height. The acoustic stimuli for the present study were the same as those presented in Avivi-Reich et al. (2020), however the Signal to Noise Ratios (SNRs) used were adjusted to accommodate for age-related or language-related changes in speech recognition.

The target sentences used in the present study were the same as those reported in Avivi-Reich et al. (2020). Target sentences were 312 syntactically-correct-but-semantically-anomalous sentences spoken by a female talker and developed by Helfer (1997). Each sentence contained three target words in sentence frames such as “A *spider* will *drain* a *fork,”* or “A *shop* can *frame* a *dog”* (target words italicized). The sentences were divided into 24 lists each comprising of 13 sentences. During the Compact-Target conditions, target sentences were presented over the front loudspeaker while the masker was either presented over all three loudspeakers to create a diffused image, or over the central loudspeaker only to create a compact image of the sound source. During the Diffuse-Target conditions, the target sentences were presented over all three loudspeakers to create a diffused target image while the masker was either presented from all three loudspeakers to create a diffused image, or over the central loudspeaker only to create a compact image of the masking sound source.

Target sentences were presented in one of three masking stimuli (Noise, Babble, Speech), as described in Avivi-Reich et al. (2020). The Noise masker was a steady-state speech-spectrum noise recorded from an audiometer (Interacoustic [Assens, Denmark] model AC5). The Babble was a 12-talker babble taken from the modified SPIN test (Bilger et al., 1984). The Speech masker was created using an additional set of semantically anomalous sentences spoken by two female talkers (315-s-long track presented in a continuous loop). The target sentences were presented at an average sound pressure of 55 dBA at the estimated center of a listener’s head. The sound pressure level of the maskers was adjusted in order to produce 4 different SNRs depending on the listener Group, Masker Type, and the Timbre Condition tested. The sound pressure was measured using a Brüel and Kjær (Copenhagen, Denmark) KEMAR dummy-head to ensure that the voltages of the sounds presented in the three loudspeaker conditions were adjusted appropriately so that the sound pressure recorded at the KEMAR head in the three-loudspeaker conditions matched the sound pressure recorded at the KEMAR head in the single loudspeaker conditions. In addition, the sound level calibrations were confirmed using a Bruel and Kjaer sound level meter (Model 2260) at the location corresponding to the approximate center of a participant’s head. However, these rigorous measuring procedures do not eliminate certain comb filtering effects which will be further addressed when discussing the results (for more details concerning comb filtering effects in these conditions, see Avivi-Reich et al., 2020).

The different SNRs used were initially chosen based on previous studies that used similar stimuli in noise (e.g., Avivi-Reich et al., 2018) and then altered according to the results of two rounds of preliminary pilot testing conducted under the present listening conditions. The SNRs used in the current study are presented in Table 1. A single list of 13 sentences was used for each of the SNR values that appear in the table.

**TABLE 1:** The values of the four Signal to Noise Ratios (SNRs) used under each condition: (1) compact targets and maskers (T_C_M_C_), 2) compact targets and diffuse maskers (T_C_M_d_), 3) diffuse targets and maskers (T_d_M_d_), and 4) diffuse targets and compact maskers (T_d_M_C_), for each of the three masker types (S, Speech; N, Noise; B, Babble), presented separately for the two experimental groups of the Young-ESL and Old-EFL participants.

Old-EFL						Young-ESL					
	
TcMc			TcMd			TcMc			TcMd		
			
S	N	B	S	N	B	S	N	B	S	N	B
10	8	–3	3	2	–10	11	6	–3	5	5	–7
4	3	–9	–3	–3	–16	5	1	–9	0	–1	–13
–2	–2	–15	–9	–8	–22	–1	–4	–15	–5	–7	–19
–8	–7	–21	–15	–13	–28	–7	–9	–21	–10	–13	–25

**TdMc**			**TdMd**			**TdMc**			**TcMd**		
			
**S**	**N**	**B**	**S**	**N**	**B**	**S**	**N**	**B**	**S**	**N**	**B**

14	11	4	10	8	1	11	9	–2	11	6	–3
8	6	–2	4	3	–5	5	4	–8	5	1	–9
2	1	–8	–2	–2	–11	–1	–1	–14	–1	–4	–15
–4	–4	–14	–8	–7	–17	–7	–6	–20	–7	–9	–21

Trials were blocked according to lists. All sentences in a list were presented at a constant SNR. In the two experimental groups (T_C_, T_d_), six participants were tested with a diffused masker (M_d_) for the first 12 lists, and then with a compact masker (M_C_) for the remaining 12. The reverse order was applied for the other six participants. Sentence lists and SNRs were counterbalanced across participants such that each list was presented at each of the 4 different SNRs an equal number of times within each group. Moreover, each list was presented in each of the four Timbre Conditions (T_C_M_C_, T_C_M_d_, T_d_M_d_, T_d_M_C_) and three Masker (Speech, Babble, Noise) combinations an equal number of times.

Before starting the experimental session, participants were given a brief explanation to become familiarized with the task. Participants were asked to repeat back the target sentence after each presentation and were scored for the correct repetition of any keyword. Performance was assessed in real-time while the session was taking place, and later by a second research assistant who listened to the participant’s recorded responses. If there was a disagreement between the online assessment and the second listener’s coding of the sentences, the two raters listened to the recording together, until they arrived at a consensus opinion. After each response by the participant, the researcher began the next presentation of the trial. Each trial began with the masker sound which was followed 1 s later by the target sentence. The masker remained on during the presentation of the target sentence, then the masker was turned off when the target sentence ended. After completing 12 lists, a short break was offered to the participants.

## Results

### Demographic Data

Table 2 presents the gender breakdown, mean age, Mill Hill test of vocabulary knowledge and Nelson-Denny test of reading comprehension results for the young English as first language Young-EFL participants (Young-EFL) in Avivi-Reich et al. (2020), and the older English as first language participants (Old-EFL), and the young English as a second language (Young-ESL) participants in this experiment. An Age Group (Young-Old) by Language Status (EFL-ESL) by Target Timbre Between-Subjects ANOVA found a significant age difference between the younger and older groups [*F*(1, 66) = 3,723., *p* < 0.001]. There were no differences in age between the EFL and ESL groups, and those participants in the Compact Target group and Diffuse Target Group. In addition, none of the interactions were significant (all *F*-values < 1).

**TABLE 2:** The gender breakdown, mean age, Mill Hill vocabulary test and Nelson-Denny reading comprehension test results for the Young-EFL (taken from Avivi-Reich et al., 2020), and for the Old-EFL and the Young-ESL participants in this experiment. SE stands for Standard Error.

Group	Gender	Age in years	Mill Hill vocabulary	Nelson-Denny reading
Young EFLs compact target	4 Male 8 Females	Mean = 21.78 SE = 0.61	Mean = 14.50 SE = 0.36	Mean = 28.33 SE = 1.15
Young ESLs compact target	3 Males 9 Females	Mean = 21.19 SE = 0.45	Mean = 9.25 SE = 1.16	Mean = 18.08 SE = 1.77
Young EFLs diffuse target	1 Male 11 Females	Mean = 20.14 SE = 0.51	Mean = 13.00 SE = 0.77	Mean = 25.83 SE = 1.71
Young ESLs diffuse target	3 Males 9 Females	Mean = 21.02 SE = 0.56	Mean = 10.00 SE = 0.72	Mean = 20.67 SE = 1.77
Old EFLs compact target	1 Male 11 Females	Mean = 72.76 SE = 1.31	Mean = 15.45 SE = 0.68	Mean = 23.83 SE = 1.80
Old EFLs diffuse target	3 Males 9 Females	Mean = 72.75 SE = 1.21	Mean = 14.92 SE = 0.87	Mean = 22.67 SE = 1.65

An Age Group (Young-Old) by Language Status (EFL-ESL) by Target Timbre Between-subjects ANOVA on Mill Hill vocabulary scores found a highly significant effect of language status [EFLs had higher vocabulary scores than ESLs: *F*(1, 66) = 26.905, *p* < 0.001], and a nearly significant effect of Age-Group [*F*(1, 66) = 3.258, *p* = 0.076] where older adults had higher vocabulary scores than younger adults. The effect of Target Timbre failed to reach significance [*F*(1, 66) < 1], and there was no evidence of an interaction between Language Status and Target Timbre [*F*(1, 66) = 2.001, *p* = 0.162] and no evidence of an interaction between Age Group and Target Timbre [*F*(1, 66) < 1].

An Age Group (Young-Old) by Language Status (EFL-ESL) by Target Timbre Between-subjects ANOVA on Nelson Denny reading scores found a highly significant effect of language status [Young EFLs had better reading comprehension scores than Young ESLs: *F*(1, 66) = 21.664, *p* < 0.001], and a significant effect of Age-Group [*F*(1, 66) = 5.358, *p* = 0.024] where younger adults had higher reading scores than older adults. The effect of Target Timbre failed to reach significance [*F*(1, 66) < 1], and there was no evidence of an interaction between Age Group and Target Timbre [*F*(1, 66) < 1] or of an interaction between Language Status and Target Timbre [*F*(1, 66) = 2.355, *p* = 0.130].

### Psychometric Functions

**FIGURE 2:**
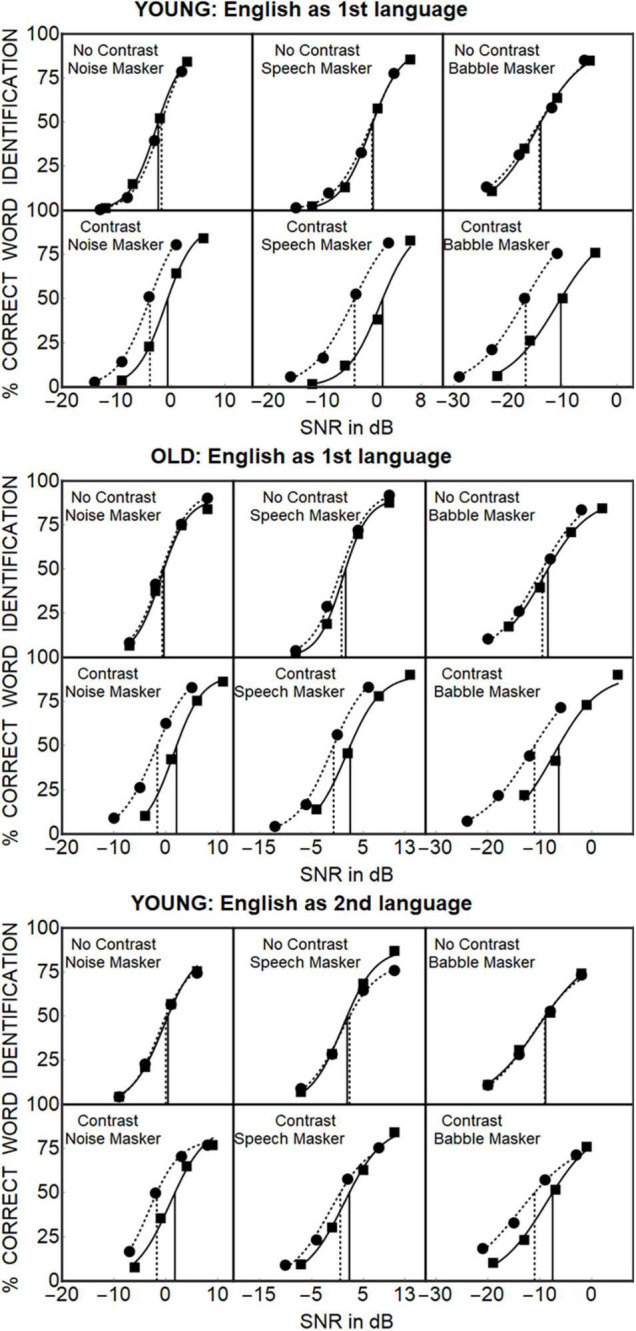
**Top.** Young-EFL participants from Avivi-Reich et al. (2020). **Center.** Old-EFL participants, this experiment. **Bottom.** Young-ESL participants, this experiment. The percentage of words correctly identified is plotted as a function of SNR for each combination of the three maskers (Noise, Speech, and Babble) with the four target-masker combinations (TcMc, TdMd, TcMd, TdMc) for the three different groups of participants. The data for the No Contrast Conditions (TcMc and TdMd) are shown separately from the data when there is a timbre contrast between target and masker (TcMd and TdMc). Circles represent the average data for the Conditions where the target was compact (Tc), squares represent the data for diffuse targets (Td). Solid lines show the psychometric functions fit to that data when the target was diffuse (Td); dotted lines show the psychometric functions fit to the data when the target was compact (Tc). The SNRs corresponding to 50% correct identification when the target is compact are indicated by the dotted vertical lines. The solid vertical lines indicate the corresponding SNRs when the target is diffuse.

Figure 2 (Top Portion) shows the percentage of correctly identified keywords for the 24 young participants whose first language was English (Young-EFLs) as a function of SNR when the masker was speech spectrum noise (left panel), two-talker speech (center panel) or 12-talker babble (right panel). Twelve of these participants were presented with compact targets (Tc) only, while the other 12 participants were presented only with diffuse targets (T_d_) (These data were adapted from Avivi-Reich et al., 2020). Psychometric functions are plotted separately for instances in which there is no contrast in timbre between the target and masker (T_C_M_C_ and T_d_M_d_), and those in which there is a timbre contrast between the target and masker (T_C_M_d_ and T_d_M_C_). Circles represent the data for the compact target (T_C_) group with squares representing the data for the diffuse target (T_d_) group. Logistic psychometric functions of the form


(1) $$y=\frac{100*a}{1+e^{-\sigma \,\left(x-\mu \right)}}$$

were fit to these data points, where the parameter *a* is restricted to the range from 0 to 1, and 100*a specifies the asymptotic value reached by the percent correct word recognition as the SNR, *x*, approaches infinity (i.e., when listening in quiet). The parameter μ denotes the value of *x* such that the percent correct word recognition reaches ½ of its asymptotic value, and σ controls the slope of the function (for a description of the fitting procedure see Supplementary Appendix 1). The 50% points on these fitted psychometric functions are indicated by the dashed vertical lines when the target speech was compact (T_C_), and solid vertical lines for when the target speech was diffuse (T_d_) and were used as estimates of the speech recognition threshold for that condition.

The center portion of Figure 2 plots the equivalent data from the 24 older participants whose first language was English (Old-EFL), while the bottom portion shows the results from the 24 participants for whom English was a second language (Young-ESL). For all three groups, when there is no timbre contrast between target and masker (T_C_M_C_ or T_d_M_d_), the psychometric functions appear to be equivalent, independent of whether the target was compact (solid circles) or diffuse (solid squares). However, when there is a contrast in timbre between target and masker (T_C_M_d_ or T_d_M_C_), the psychometric functions for the conditions in which the target is diffuse (filled squares) are shifted to the right with respect to conditions in which target is compact (filled circles) in all three groups. There are, however, indications that Target Timbre, Masker Type and Language Status affects the 50% thresholds of the psychometric functions, as well as their slopes. First, Figure 2 shows that thresholds are lowest for the Young-EFL group when compared to the other two groups. Second, when there is a timbre contrast between target and masker, the degree of separation between the psychometric functions for the T_C_M_d_ and the T_d_M_C_ conditions appears to depend on both their Linguistic Group, and the type of Masker (Noise, Babble, or Speech). It should also be noted that when there is no timbre contrast between target and masker, the effect of the signal-to-masker ratio appears to be the same independent of whether the target is compact or diffuse, as long as the masker timbre is the same as the target timbre.

To confirm these visual impressions, statistical analyses were conducted on individual participants with respect to the three parameters of the psychometric function. Specifically, psychometric functions were fit to all individuals in order to obtain individual estimates of the threshold, μ, the slope parameter, σ, and the asymptotic value (*a*) of the psychometric functions. We then conducted a 3 Group (Young-EFLs, Old-EFLs, Young-ESLs) × 2 Target Timbres (T_C_ vs. T_d_) × 3 Masker Types (Noise, Babble, Speech) × 2 Masker Timbre conditions (M_C_ vs. M_d_) ANCOVA with Participant Group, and Target Timber as between-subjects factors and Masker Type and Masker Timbre as within-subject factors, with vocabulary and reading comprehension as covariates, for thresholds and slopes, following the procedure recommended by Schneider et al. (2015). The results of this analysis of variance are shown in Supplementary Table 1. All four main effects (Masker Type, Masker Timbre, Target Timbre, and Group were highly significant (*p* < 0.001, for the main effects of all four factors). There were also 3 three-way interactions that were significant (MaskerType × TargetTimbre × Group, *p* = 0.001; MaskerType × MaskerTimbre × TargetTimbre, *p* = 0.002; MaskerType × MaskerTimbre × Group, *p* = 0.01), and 1 two-way interaction (MaskerType × Group, *p* = 0.005). None of the other interaction effects were significant. In addition, there was no evidence that the two covariates affected performance. Hence, none of the subsequent analyses involved the covariate measures.

Because Figure 2 suggests that Target Timbre has a negligible effect on thresholds when the timbre of the target matches the timbre of the masker, we conducted two additional analyses to determine the sources of the interaction effects found in the omnibus ANOVA. First, we conducted a three Group (Young-EFLs, Old-EFLs, Young-ESLs) × two-target timbres (T_C_ & T_d_) × three Masker Types (Noise, Speech, and Babble) ANOVA only for the conditions in which the timbre of the masker matched that of the target, with Group and Target Timbre as between-subjects factors, and Masker Type as a within-subject factor. Supplementary Table 2 shows that when the target’s timbre matches that of the masker, none of the effects involving the target’s timbre are significant. Hence, the source of any of the interaction effects involving the target’s timbre in the omnibus ANOVA are restricted to conditions in which there is a mismatch between the target’s timbre and the masker’s timbre.

**FIGURE 3:**
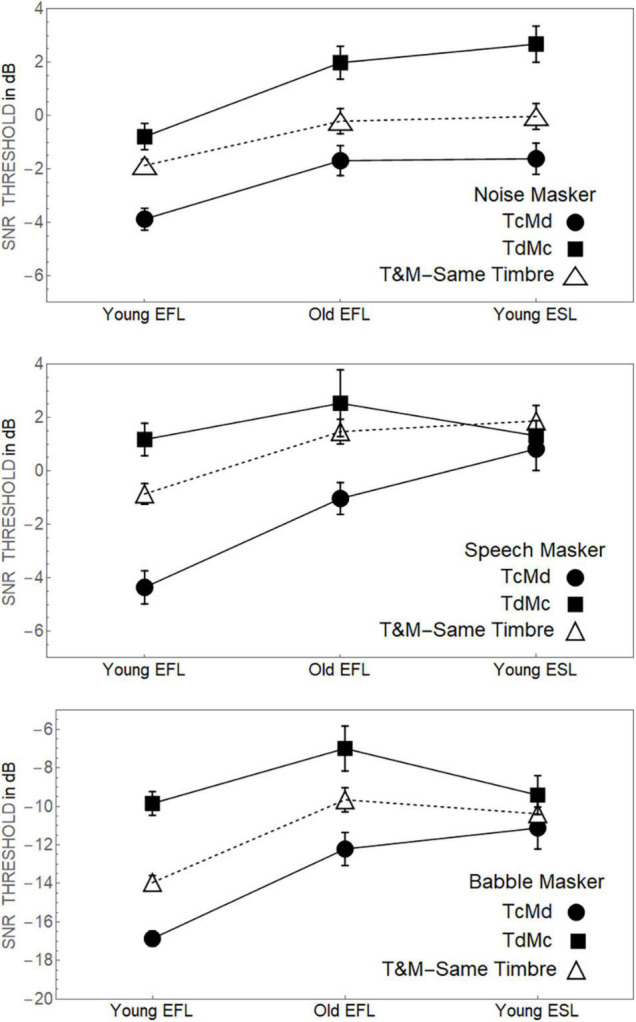
**Top panel.** Fifty percent thresholds when the masker was speech spectrum noise for the three groups listening to sentences where the target was either compact or diffuse with the masker having the opposite timbre. **Middle Panel.** The equivalent data when the participants were listening to the sentences when the background is competing speech. **Bottom Panel.** The equivalent data when the participants were listening to the sentences when the background is babble. Standard error bars are shown.

A comparable analysis (see Supplementary Table 3) limited to when there was a mismatch between the target’s timbre and the masker’s timbre, however, found a significant three-way interaction between Target Timbre, Masker Timbre, and Group (*p* < 0.001). To identify the source of this three-way interaction, Figure 3 plots how the thresholds for both T_C_M_d_ and T_d_M_C_ conditions change as a function of Group, separately for the Noise, Speech and Babble Maskers. Also shown are the average thresholds for the two conditions in which the target timbre matched the masker timbre (average of T_C_M_C_ and T_d_M_d_ thresholds). This figure indicates that for Noise maskers the separation between the T_C_M_d_ and T_d_M_C_ thresholds remains constant across the three Groups. However, for Speech and Babble Maskers, the advantage held by compact targets is severely diminished in the Young-ESL group compared to the Young-EFL group. Subsequent analyses in Supplementary Appendix 2 shows that if the Young-ESL group is excluded from the analysis, there is no indication of an interaction between the two remaining EFL groups (Young-EFLs and Old-EFLs) and target timbre. However, when considering only young adults, there is a highly significant interaction between their linguistic status (EFL vs. ESL) and target timbre, highlighting the importance of the language status of people in a complex acoustic environment. An examination of Figure 3 suggests, for young-ESL adults in both Babble and Speech Maskers, that the thresholds were essentially equivalent, for all combinations of target and masker timbre. Pairwise comparisons of the young-ESL thresholds among the four combinations of target and masker (T_C_M_C_, T_C_M_d_, T_d_M_C_, T_d_M_d_) failed to find any significant differences in threshold values when the masker was Babble for a Type 1 error of 0.05 (after applying a Bonferroni correction for the six comparisons). For the equivalent comparisons of Young-ESL thresholds in Speech, only one of the comparisons was significant (T_C_M_C_ vs. T_C_M_d_). However, the difference in threshold between these two timbre conditions in the Young-ESL listeners (1.8 dB) was much smaller than the difference in the same two timbre conditions for the Young-EFL listeners (3.1 dB).

**FIGURE 4:**
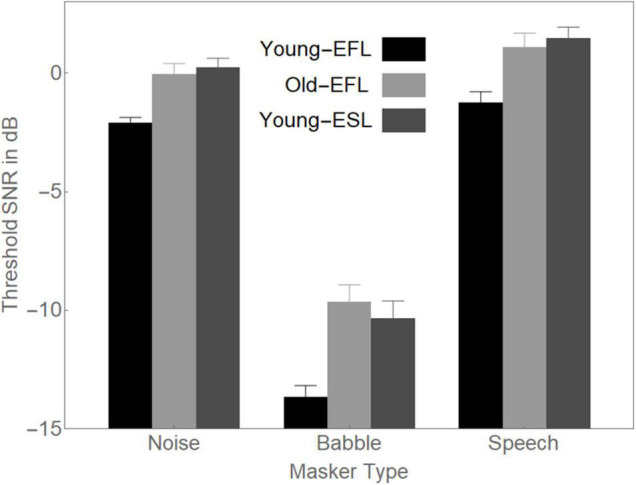
Average 50% thresholds in Noise, Babble, and Speech for the three groups of participants. Error bars are standard errors of the means.

To determine the source of the two-way interaction in the omnibus ANOVA between Group and Masker Type when there is a mismatch between Target Timbre and Masker Timbre, in Figure 4, we plotted, for each of the Masker Types, the average thresholds for each of the Groups.

In Figure 4, the difference between Noise thresholds and Babble thresholds appears to be larger for Young-EFLs (11.6 dB) than it is for either Old-EFLs (9.6 dB) or Young-ESLs (10.6 dB). Similarly, the difference between Speech thresholds and Babble thresholds appears to be larger for Young-EFLs (12.4 dB) than it is for either Old-EFLs (10.7 dB) or Young-ESLs (11.8 dB). To confirm that the interaction between Masker Type and Group is due to the larger separation in the Young-EFL group between Noise and Babble, and between Speech and Babble than the comparable comparisons in the other two Groups, a separate ANOVA was conducted that excluded the Babble Masker condition. When the Babble Masking condition was excluded, there was no evidence of an interaction between Group and Masker Condition [*F*(2, 66) = 0.270, *p* > 0.5]. Hence, the two-way interaction between Masker Type and Group appears to be due to the very low threshold in Babble that is found in the Young-EFL participants.

### Slopes of the Psychometric Functions

We also conducted an ANOVA on the slopes of the individual psychometric functions with Target Timbre and Group as between-subjects factors and Masker Type and Masker Timbre as within-subject factors. The only factor that significantly affected the slopes of the psychometric functions was the Masker Type [*F*(2, 132) = 8.711, *p* < 0.001]. As Figure 2 suggests the slopes for Speech (Mean = 0.49) and for Noise (Mean = 0.41) are greater than those for Babble (Mean = 0.23). Pairwise *T*-test indicate that the difference in slopes between Noise and Speech were not significant [*T*(71) = –1.08, *p* = 0.284], but the differences in slopes between Noise and Babble [*T*(71) = 8.87, *p* < 0.0001], and Speech and Babble [*T*(71) = 3.21, *p* = 0.002] were significant (for more information see Supplementary Table 4).

### Asymptotes of the Psychometric Functions

The mean asymptote (*a*) of the psychometric functions for the three linguistic groups were: (1) Young-EFLs (0.94); (2) Old-EFLs (0.92); and (3) Young-ESLs (0.84). A *T*-test of the difference between Young-EFL and Old-EFL asymptotes was not significant [*T*(46) = –1.17, *p* = 0.25]. A *T*-test of the difference between Young-EFL and Young-ESL asymptotes was significant [*T*(46) = –4.33, *p* < 0.0001], as was the difference between Old-EFL and Young-ESL asymptotes [*T*(46) = –3.14, *p* = 0.003]. Hence, asymptotes for young and old native listeners were comparable, but both of these groups had significantly higher asymptotic values than did the Young-ESL group.

## Discussion

In the current study, three different masker types were used (Noise, Babble and Speech) to test the effect of sound source diffuseness on speech recognition in Young-ESL and Old-EFL listeners and compare their performance to that of the Young-EFL listeners previously tested (see Avivi-Reich et al., 2020). The results showed that for all three groups, when there is no timbre contrast between target and masker (T_C_M_C_ or T_d_M_d_), the psychometric functions appear to be equivalent, independent of whether the target was compact or diffuse. In other words, the Target Timbre has a negligible effect on thresholds when the timbre of the target matches the timbre of the masker (T_C_M_C_ or T_d_M_d_). These findings are similar to what was previously found in Young-EFL listeners (Avivi-Reich et al., 2020). However, when there is a contrast in timbre between target and masker (T_C_M_d_ or T_d_M_C_), a significant separation between the T_C_M_d_ and T_d_M_C_ thresholds is evident in all three groups when the masker is Noise. Interestingly, for Speech and Babble Maskers, the advantage held by compact targets is severely diminished in the Young-ESL group compared to two EFL groups (young and old). Indeed thresholds for all four conditions (T_C_M_C_, T_C_M_d_, T_d_M_d_, T_d_,M_C_) appear to be quite similar (see Figure 3). This would suggest, that, in the presence of informational masking, Young-ESLs are unable to use timbre differences to attend to and process the target speech. These results indicate that listeners, whose linguistic status differs, respond to timbre differences differently depending on masker type. Young-EFLs and Old-EFLs appear to derive equivalent benefits from timbre differences between targets and maskers. Thus, it seems that while Old-EFLs generally need more favorable SNRs compared to Young-EFLs to correctly recognize speech in the presence of competing sounds, the different diffuseness levels between targets and maskers seem to affect both EFL age groups similarly.

In addition, a two-way interaction between Masker Type and Group was found, which appears to be due to the larger separation between Noise and Babble and between Speech and Babble thresholds in the Young-EFL group than in the other two Groups. In other words, the Young-EFL listeners, who overall had better (lower) speech recognition thresholds compared with the other two groups, did exceptionally better when the masker was Babble. Hence, when there is a babble of indistinguishable voices, Young-EFL listeners have exceptionally low thresholds compared to either Old-EFL listeners or young-ESL listeners.

Two possible reasons were previously suggested and discussed (Avivi-Reich et al., 2020) as to why listeners may find auditory scenes in which the target is compact and the masker is diffused more favorable than when there is no such timbre contrast between the sound sources, while they seem to find the opposite configuration (Target is diffuse and Masker is compact) less favorable than listening in an auditory scene with no timbre contrast. The first is that compact sound sources with a precise location may attract the listener’s attention, giving the compact sound source a certain advantage, which could either serve speech recognition when the speech sound is compact, or potentially increase the interference when the irrelevant competing sound is the compact one. The second possible explanation is that the pattern of results found is consistent with what would be expected when taking into consideration the comb-filtering effects that occur when a sound source is played over multiple loudspeakers vs. when it is played over a single loudspeaker only. When the same sound is played over spatially separated loudspeakers, it will arrive at the ear of the listener at slightly different times. These delays result in some frequencies being enhanced, while others are canceled, producing peaks and troughs in the sound spectrum at the ears. Hence, when the masker is diffuse, there will be peaks and troughs in the spectrum of masker. If the listener can attend to and integrate the information in the speech target falling into the troughs of the masker, we might expect to find lower thresholds when the masker is diffuse and the target is compact. For a fuller explanation (see Avivi-Reich et al., 2020).

With these two possible explanations in mind, we would like to address the primary question raised by the current findings. First, why would all three groups (Young-EFLs, Old-EFLs, Young-ESLs) in the Noise condition, have lowest thresholds when the target is compact and the masker is diffuse (T_C_M_d_) and highest thresholds when the target is diffuse and the masker compact (T_d_M_C_) with the T_d_M_d_ and T_C_M_C_ conditions falling midway between the two? Second, why do the Young-EFL and Old-EFL listeners show this same pattern when the Masker is Babble or Speech, but not the young-ESL listeners, who perform equivalently in all four timbre conditions? To answer these questions, we will need to consider the ways in which the Noise masker is different than Babble and Speech, as well as the differences between EFL-listeners and ESL-listeners.

Noise, Babble and Speech maskers are all expected to cause interference resulting in a greater difficulty to recognize speech. However, the level of processing at which this interference occurs is likely to differ among masker types. All three masker types used in the current study (Noise, Babble, Speech) activated regions along the basilar membrane that undoubtedly overlap with those activated by the target speech. Such overlap energetically interferes with the encoding of the target speech signal causing peripheral or energetic masking (Pollack, 1975). When the masker used was speech from one or more talkers (Speech or Babble), it likely also interfered with the linguistic and semantic processing of the target speech causing informational masking as well as energetic masking (for a review, see Durlach et al., 2003; Freyman et al., 2004; Schneider et al., 2007, 2010; Kidd et al., 2008). Mattys et al. (2009) divided informational masking interference into three categories: (1) The effects of the masker competing for attention including the cost of inhibiting information coming from the competing speech; (2) interference from a known language when the masker itself is intelligible and meaningful, thereby leading to lexical-semantic interference; (3) additional cognitive load associated with the processing resources required when listeners need to divide their attention between the target and the masker. The three types of maskers used in the current study differ in the levels of energetic and informational making they cause. While the Noise masker generates relatively consistent energetic masking across a wide range of frequencies, it contains no verbal information and therefore is not expected to generate informational masking. Babble and Speech, however, lead to intensity fluctuations over time creating energetic peaks and troughs. In addition, it is reasonable to expect that due to the greater resemblance between the target speech and a speech masker (Speech or Babble), compared to that found between the target speech and a noise masker, stream segregation will be more difficult to obtain when the masker is speech or babble.

Several speech perception studies have included different types of maskers in order to study the effect type of masker may have on the extent to which listeners experience release from masking when provided with an assisting cue that could enhance speech perception (e.g., Freyman et al., 2004; Ezzatian et al., 2010; Mattys et al., 2010; Avivi-Reich et al., 2018). Their findings have shown that the amount of release provided by a particular manipulation differed depending on the type of masker that was presented. Interestingly, in several previous studies that examined spatial cues (such as location and spatial separation cues), the release from masking generally increases with the informational content of the masker (e.g., Arbogast et al., 2002; Ezzatian et al., 2010). For example, Ezzatian et al. (2010) asked young-EFL and young-ESL listeners to repeat sentences that were presented to them in the presence of either Noise, Babble or competing Speech, when the target and masker were co-located vs. when there was spatial separation between the two. Their results showed that the amount of release from masking due to spatial separation is larger when the masker is speech rather than noise. In addition, young-EFL and young-ESL listeners benefited equally from perceived spatial separation. This pattern of results resembles what was found for the Young-EFL listeners in the previous experiment, but somewhat contradicts the pattern found in the Young-ESL listeners.

Figure 3 suggests that for Young-ESL participants listening in the presence of a Babble or a Speech masker, thresholds for target speech recognition appear to be independent of the timbres of the target speech and the masker, and depend solely on the SNR (the one exception is the T_C_M_C_ vs. T_C_M_d_ comparison for the Speech Masker). We might expect such a result if the Young-ESL listeners were unable to take advantage of differences in timbre between target and masker. If that were the case, then thresholds would depend solely on the ratio of speech energy to masker energy.

Why might this be the case? The results from the conditions where the masker was Noise clearly indicates that speech recognition is sensitive to timbre differences between the target speech and masker for Young-ESL listeners. Hence, they can use these cues in some difficult listening situations. If that is the case, why do they not use these cues when the masker is Babble or Speech? One possibility is that in order to benefit from timbre differences, the listener has to allocate attentional resources to basic auditory processes in order to extract a benefit from timbre differences. In a previous paper, we pointed out that a diffuse masker produces troughs in the spectrum of the masker. If the listener is able to focus attentional resources in the frequency regions corresponding to the troughs and integrate the information from these troughs to extract the speech signal (Scharf et al., 1987), then we would expect lower speech recognition thresholds when the target is compact, and the masker is diffuse. The Young-ESL listeners can clearly do this when the masker is Noise, but not when the masker is Babble or Speech.

The reason for this difference may reside in the additional attentional resources that need to be deployed by second language listeners when the masker is either babble or speech. Second language listeners are found to have lower performance than listeners listening to their first language on a number of auditory speech-perception measures (Mayo et al., 1997; Bradlow and Pisoni, 1999; Meador et al., 2000; Bradlow and Bent, 2002; Cooke et al., 2008; Rogers and Lopez, 2008; Ezzatian et al., 2010; Avivi-Reich et al., 2014, 2015). Second language listeners tend to experience interference from their first language knowledge when listening to speech in their second language (Nábělek and Donahue, 1984; Bradlow and Pisoni, 1999; Cutler, 2001). The speech perception differences found between first and second language listeners could be due, in part, to incomplete acquisition of the acoustic–phonetic characteristics of the second language (e.g., Florentine, 1985; Mayo et al., 1997), which might lead to a reduced ability to correctly recognize the phonemes in one’s second or third language (Bradlow and Pisoni, 1999; Meador et al., 2000). In addition, in second language listeners the semantic and linguistic processes in their second language may not be completely differentiated from those in their first (Kroll and Steward, 1994). Thus, this cross-linguistic interference could be a result of phonetic, phonemic and or phonotactic knowledge transfers (e.g., Polka, 1991, 1992). When both the target and the masker contain speech in their second language, second language listeners might find speech recognition to be especially difficult. The overlap between the two linguistic systems could result in greater competition as both systems are activated by more than a single incoming verbal stream. Hence, the degree and extent to which second language listeners must engage attentional and knowledge-driven processes (e.g., vocabulary and linguistic knowledge) to facilitate speech perception could differ from the pattern of engagement in first language listeners. This additional load may leave them with inadequate attentional resources to focus attention on particular regions along the basilar membrane.

If indeed the cause for the interaction found between the listeners’ linguistic status and the effect of timbre contrast on speech recognition is due to greater draw on scarce attentional resources, it is reasonable to assume those could be captured by listening effort measurements. Thus, it is recommended that future studies use listening effort measures, such as pupilometry or dual-task, to further examine speech perception and the connection between linguistic experience and listening effort under different timbre conditions. The relationship between resource demand and listening-effort has been established by numerous studies (e.g., Koelewijn et al., 2012; Zekveld et al., 2014; Pichora-Fuller et al., 2016; Gagné et al., 2017; Tangkhpanya et al., 2019), incorporating a measure of effort would allow us to better understand the difficulties listeners might experience when listening to their second language in complex and acoustically amplified listening environment and contribute to the development of more accommodating sound amplification.

Why then are the Young-ESL listeners able to benefit as much from spatial separation as Young-EFL listeners? The reason might be that locating the azimuth positions of auditory objects is an automatic process, one that does not require attentional resources. The binaural system is exquisitely sensitive to time of arrival differences of a sound to the two ears, as well as differences in intensity. Time of arrival differences are coded at the level of the cochlear nucleus and are an intrinsic part of the auditory signal processed by higher-order brain structures. As such, they most likely do not require attentional resources to code and utilize these time of arrival differences. Timbre differences, however, most likely require attention to be focused on particular spectral areas. A number of studies have shown that when attention is focused on a particular region of the spectrum, the detection of a signal in that region is dramatically improved, suggesting that frequency-selective attention involves the operation of a “listening band,” centered on the attended frequency (Scharf et al., 1987; Degerman et al., 2006; Riecke et al., 2017). Hence, if a listener could focus her or his attention on particular spectral regions, and integrate information across these regions, they could take advantage of the comb filtering provided by a diffuse masker. However, attentional selection has been characterized as a pool of attentional resources from which resources can be allocated to current tasks until the pool is exhausted (Kahneman, 1973; Lavie, 2005). Thus, if the attentional resources of the Young-ESL listeners were fully deployed at the lexical and semantic levels of processing, they might not have the resources to benefit from the increased signal-to-noise ratios that would be present in the troughs of the spectrum associated with a diffuse masker.

In summary, the results of the current study, which examines the effects of sound diffuseness levels on speech recognition in Young-ESL and Older-EFL listeners using three types of maskers (Noise, Babble, Speech) were compared to the results previously found in Young-EFLs. The comparison uncovered a significant difference in the timbre contrast effect found in the two EFL groups vs. the ESL group. While the two EFL groups demonstrated a benefit from such timbre contrast when the target was compact in the presence of all three masker types, the ESL group demonstrated improved speech recognition only when the diffused masker was Noise. A possible explanation as to why this three-way interaction was found statistically significant was suggested based on the listeners’ linguistic experience, the interference caused by energetic vs. informational masking, and the explanations that were previously provided to explain the timbre contrast effects that were found (Avivi-Reich et al., 2020). The current study joins our previous study to form what we believe to be the only systematic investigation of sound diffuseness effect. The two studies together depict sound diffuseness level as an acoustic variable that could play a significant role in speech recognition, and its overall effect is dependent on variables such as the type of masker in which the target speech is presented and the linguistic experience of the listener. As the use of amplification becomes more common in both public and private listening environments, it is important to continue investigating the possible effects of using multiple loudspeakers on the speech perception of a variety of potential listeners.

## Data Availability Statement

The raw data supporting the conclusions of this article will be made available by the authors, without undue reservation.

## Ethics Statement

The studies involving human participants were reviewed and approved by the Ethics Review Board of the University of Toronto. The patients/participants provided their written informed consent to participate in this study.

## Author Contributions

BS and MA-R conceived and planned the experiments and interpreted the results. MA-R and RKS carried out the experiments. BS took the lead in statistically analyzing the data. MA-R took the lead in writing the manuscript. All authors provided critical feedback and helped shape the research, analysis, and manuscript.

## Conflict of Interest

The authors declare that the research was conducted in the absence of any commercial or financial relationships that could be construed as a potential conflict of interest.

## Publisher’s Note

All claims expressed in this article are solely those of the authors and do not necessarily represent those of their affiliated organizations, or those of the publisher, the editors and the reviewers. Any product that may be evaluated in this article, or claim that may be made by its manufacturer, is not guaranteed or endorsed by the publisher.
