# Supplementary material for: Do Age and Linguistic Status Alter the Effect of Sound Source Diffuseness on Speech Recognition in Noise?
Source: Front Psychol. 2022 Mar 15;13:838576. doi: 10.3389/fpsyg.2022.838576 (PMC8965325; doi:10.3389/fpsyg.2022.838576)
Supplement: Supplementary file 1 [file Data_Sheet_1.docx]

**Appendix 1.**

A preliminary visual examination of plots of percentage correct identification in the Young-ESL participants strongly suggested that the asymptotes of psychometric functions fit to the data were likely to plateau at significantly less than 100% as the SNR increased (SNR -> Infinity). This might be expected in this group since they do not have the same degree of command of the English language as do the EFL participants, as indicated both by the Mill Hill vocabulary scores, and the Nelson Denny Reading Comprehension Test (see Results section), especially because the semantically anomalous sentences used as targets are mostly devoid of contextual support. Hence, we decided to fit logistic psychometric functions where the asymptote could be at less than 100%. This would allow for the possibility that the ESL participants’ command of the English language was somewhat less than that of the Young-EFL and Old-EFL participants.

Because the justification for doing this was the idea that the Young-ESLs might not reach 100% correct identification even with an infinite SNR (no noise), we expected the asymptotic value in a no-noise condition for an individual to be the same in all six conditions for the 12 Young-ESLs who were tested with a compact Target (Noise-T_c_M_c_, Noise-T_c_M_d_, Babble-T_c_M_c_, Babble-T_c_M_d_, Speech-T_c_M_c_, Speech-T_c_M_d_), as well as for the 12 Young-ESLs who were tested with a diffuse Target (Noise-T_d_M_c_, Noise-T_d_M_d_, Babble-T_d_M_c_, Babble-T_d_M_d_, Speech-T_d_M_c_, Speech-T_d_M_d_). For each participant, we started the fitting process by assuming that *a* = 1 in Equation 1, which would result in 100% correct identification in quiet (SNR = Infinity). We then followed the procedure used in Yang, Chen, Huang, Wu, Wu, Schneider, Li (2007), to find the best-fitting values of $\mu$ and $\sigma$ with the asymptote fixed at 100% (*a=1*) in each of the six conditions for that participant. This procedure finds the values of $\mu$ and $\sigma$ that minimizes the Pearson’s Chi Square between the predicted and obtained number of correct identifications in that condition. The resulting six Chi Square values (one for each of the six conditions) were then summed to compute a composite Chi Square for that participant assuming that *a* = 1. Then the value of *a* was reduced by .01, and the procedure repeated for that participant producing a composite Chi Square value for *a* = .99. The value of *a* then was reduced in steps of .01 to find the *a* value that produced the minimum composite Chi Square. The value of *a* associated with the minimum composite Chi Square for a participant, when multiplied by 100, was used to estimate the asymptotic performance expected of that individual in quiet.

To be consistent, we then fitted individual asymptotes for the Young-EFL and Old-EFL individuals. However, because the Young-EFL participants in this study were the same as the Young-EFL participants in Avivi-Reich et al. (2020), we needed to check whether allowing individual asymptotes to be fitted to each participant would alter the description of the thresholds reported in that study in any significant way. When we made this comparison, we found that all of the factors that significantly affected the thresholds in Avivi-Reich et al. also affected the thresholds when individual asymptotes were fit to the individuals in that study. However, when individual asymptotes were fit to the data, the interaction effect between Masker Type and Masker Timbre on the thresholds, which was previously insignificant (p = .07), now became significant (p = .027). Apparently, fitting individual asymptotes affected the 50% thresholds to the extent that a nearly significant interaction between Masker Type and Masker Timbre reached significance. A comparison between the psychometric functions obtained in Avivi-Reich et al., where all individuals were assumed to have an asymptotic value of 100% (see Figure 3 in Avivi-Reich et al.), with those found when individual asymptotes were fitted to the data (Figure 2, this manuscript), indicates that the psychometric functions were comparable independent of whether or not individual asymptotes were fit to the data.
