# Supplementary material for: Do Age and Linguistic Status Alter the Effect of Sound Source Diffuseness on Speech Recognition in Noise?
Source: Front Psychol. 2022 Mar 15;13:838576. doi: 10.3389/fpsyg.2022.838576 (PMC8965325; doi:10.3389/fpsyg.2022.838576)
Supplement: Supplementary file 2 [file Data_Sheet_2.docx]

**Appendix 2**.

A three-way ANOVA (see Table A3) conducted on the conditions in which there is a mismatch between the target’s timber and masker’s timbre found main effects of Target Timbre, Group, and Masker Type, along with the 3-way interaction shown in Figure 3. The source of this 3-way interaction, which appears to be due to the convergence of the T_c_M_d_ and T_d_M_c_ thresholds in the Young-ESL subjects when the masker is Babble or Speech, can be evaluated by first dropping the Young-ESL group from the analysis. The resulting, reduced 3-way ANOVA with Target Timbre, Masker Type and the two remaining EFL groups (Young EFLs, Old EFL) as factors yielded significant effects of: 1) Masker Type (F[2,88] = 631.491, p < .001); 2) Group (F[1,44] = 21.972, p < .001); 3) Target Timbre (F[1,44] = 59.016, p < .001); and 4) a Masker Type × Target Timbre interaction (F[2,88] = 7.774, p = .001). Notably, there were no interactions involving the two remaining Groups. Hence, the three-way interaction shown in Figure 3 can be attributed to the inclusion of the English-as-a-second language group.

When we restrict the Group to young adults differing in their language status (EFL, ESL) the pattern of results is quite different. There were significant effects of 1) Masker Type (F[2,88] = 628.43, p < .001); 2) Group (F[1,44] = 28.077, p < .001); 3) Target Timbre (F[1, 44] = 46.367); 4) a two-way interaction between Group and Target Timbre (F[1,44] = 7.922, p < .007; and 5) a three-way interaction between Group, Masker Type, and Target Type (F[2,88] = 12.652, p < .001). The three-way interaction can be attributed to the fact that the separation between the average thresholds in T_c_M_d_ versus T_d_M_c_ conditions in the ESL group failed to reach significance in the Babble (T[22] = 1.15, p = .261) and Speech maskers (T[22] = .5, p > .5) conditions, whereas it was highly significant in the Noise masker (T[22] = 4.81, p < .0001) and for all three maskers in the EFL group (p < ,0001, for all three maskers).
