# Supplementary material for: Do Age and Linguistic Status Alter the Effect of Sound Source Diffuseness on Speech Recognition in Noise?
Source: Front Psychol. 2022 Mar 15;13:838576. doi: 10.3389/fpsyg.2022.838576 (PMC8965325; doi:10.3389/fpsyg.2022.838576)
Supplement: Supplementary file 3 [file Data_Sheet_3.docx]

Table A1. ANCOVA on 50% thresholds.

FIXED EFFECTS: WITHIN-SUBJECTS

| Source | df | Mean Square | F | Sig. | Partial $\eta^{2}$ |
| --- | --- | --- | --- | --- | --- |
| MaskerType | 2 | 5970.33 | 1674.52 | <.001 | .962 |
| MaskerType×TargetTimbre | 2 | 8.369 | 2.347 | .100 | .034 |
| MaskerType×Group | 4 | 13.736 | 3.853 | .005 | .105 |
| MaskerType×TargetTimbre×Group | 4 | 16.628 | 4.664 | .001 | .124 |
| Error (Masker Type, ANOVA) | 132 | 3.565 |  |  |  |
| MaskerTimbre | 1 | 343.089 | 55.499 | <.001 | .457 |
| MaskerTimbre×TargetTimbre | 1 | 1.401 | .227 | .636 | .003 |
| MaskerTimbre×Group | 2 | 9.528 | 1.541 | .222 | .045 |
| MaskerTimbre×TargetTimbre×Group | 2 | .231 | .037 | .963 | .001 |
| Error (MaskerTimbre, ANOVA) | 66 | 6.182 |  |  |  |
| MaskerType×MaskerTimbre | 2 | 2.507 | 1.493 | .228 | .022 |
| MaskerType×MaskerTimbre×TargetTimbre | 2 | 11.031 | 6.570 | .002 | .091 |
| MaskerType×MaskerTimbre×Group | 4 | 5.810 | 3.461 | .010 | .095 |
| MaskerType×MaskerTimbre×TargetTimbre×  Group | 4 | 2.199 | 1.310 | .270 | .038 |
| Error (MaskerType×MaskerTimbre, ANOVA) | 132 | 1.679 |  |  |  |

FIXED EFFECTS: BETWEEN SUBJECTS

| Source | df | Mean Square | F | Sig | Partial $\eta^{2}$ |
| --- | --- | --- | --- | --- | --- |
| TargetTimbre | 1 | 458.070 | 19.960 | <.001 | .232 |
| Group | 2 | 376.080 | 16.387 | <.001 | .332 |
| TargetTimbre×Group | 2 | 65.912 | 2.872 | .064 | .080 |
| Error (ANOVA) | 66 | 22.950 |  |  |  |

COVARIATE EFFECTS: WITHIN-SUBJECT

| Source | df | Mean Square | F | Sig. | Partial $\eta^{2}$ |
| --- | --- | --- | --- | --- | --- |
| MaskerType×MHcentered | 2 | 6.299 | 1.866 | .159 | .028 |
| MaskerType×NDcentered | 2 | 9.579 | 2.837 | .062 | .042 |
| Error(MaskerType, ANCOVA) | 128 | 3.376 |  |  |  |
| MaskerTimbre×MHcentered | 1 | .57 | .090 | .766 | .001 |
| MaskerTimbre×NDcentered | 1 | .010 | .002 | .968 | .000 |
| Error(MaskerTimbre, ANCOVA) | 64 | 6.362 |  |  |  |
| MaskerType×MaskerTimbre×MHcentered | 2 | .808 | .471 | .626 | .007 |
| MaskerType×MaskerTimbre×NDcentered | 2 | .623 | .363 | .696 | .006 |
| Error (MaskerType×MaskerTimbre, ANCOVA) | 128 | 1.716 |  |  |  |

COVARIATE EFFECTS: BETWEEN SUBJECTS

| Source | df | Mean Square | F | Sig. | Partial $\eta^{2}$ |
| --- | --- | --- | --- | --- | --- |
| MHcentered | 1 | 71.339 | 3.619 | .062 | .054 |
| NDcentered | 1 | 62.271 | 3.159 | .080 | .047 |
| Error (ANCOVA) | 64 | 19.712 |  |  |  |

Table A2. ANOVA on 50% thresholds for instances in which target timbre matched masker timbre.

FIXED EFFECTS: WITHIN-SUBJECTS

| Source | df | Mean Square | F | Sig. | Partial $\eta^{2}$ |
| --- | --- | --- | --- | --- | --- |
| MaskerType | 2 | 3156.47 | 1712.13 | <.001 | .963 |
| MaskerType×TargetTimbre | 2 | 1.049 | .569 | .568 | .009 |
| MaskerType×Group | 4 | 11.946 | 6.480 | <.001 | .164 |
| MaskerType×TargetTimbre×Group | 4 | 1.684 | .914 | .458 | .027 |
| Error (Masker Type, ANOVA) | 132 | 1.844 |  |  |  |

FIXED EFFECTS: BETWEEN SUBJECTS

| Source | df | Mean Square | F | Sig | Partial $\eta^{2}$ |
| --- | --- | --- | --- | --- | --- |
| Group | 2 | 179.595 | 11.823 | <.001 | .264 |
| TargetTimbre | 1 | 4.147 | .273 | .603 | .004 |
| TargetTimbre×Group | 2 | 32.332 | 2.129 | .127 | .061 |
| Error (ANOVA) | 66 | 15.190 |  |  |  |

Table A3. ANOVA on 50% thresholds for instances in which target timbre and masker timbre differed.

FIXED EFFECTS: WITHIN-SUBJECTS

| Source | df | Mean Square | F | Sig. | Partial $\eta^{2}$ |
| --- | --- | --- | --- | --- | --- |
| MaskerType | 2 | 2824.90 | 830.637 | <.001 | .926 |
| MaskerType×TargetTimbre | 2 | 9.827 | 2.890 | .059 | .042 |
| MaskerType×Group | 4 | 3.989 | 1.173 | .326 | .034 |
| MaskerType×TargetTimbre×Group | 4 | 20.754 | 6.103 | <.001 | .156 |
| Error (Masker Type) | 132 | 3.401 |  |  |  |

FIXED EFFECTS: BETWEEN SUBJECTS

| Source | df | Mean Square | F | Sig | Partial $\eta^{2}$ |
| --- | --- | --- | --- | --- | --- |
| Group | 2 | 196.716 | 14.110 | <.001 | .300 |
| TargetTimbre | 1 | 797.012 | 57.168 | <.001 | .464 |
| TargetTimbre×Group | 2 | 43.108 | 3.092 | .052 | .086 |
| Error | 66 | 13.941 |  |  |  |

Table A4. ANOVA on slopes ($\sigma$).

FIXED EFFECTS: WITHIN-SUBJECTS

| Source | df | Mean Square | F | Sig. | Partial $\eta^{2}$ |
| --- | --- | --- | --- | --- | --- |
| MaskerType | 2 | 2.510 | 8.711 | < .001 | .117 |
| MaskerType×TargetTimbre | 2 | .145 | 0.504 | .605 | .008 |
| MaskerType×Group | 4 | .470 | 1.630 | .170 | .047 |
| MaskerType×TargetTimbre×Group | 4 | .170 | .591 | .670 | .018 |
| Error (Masker Type) | 132 | .288 |  |  |  |
| MaskerTimbre | 1 | .293 | .763 | .386 | .011 |
| MaskerTimbre×TargetTimbre | 1 | .491 | 1.279 | .262 | .119 |
| MaskerTimbre×Group | 2 | .581 | 1.515 | .227 | .044 |
| MaskerTimbre×TargetTimbre×Group | 2 | .295 | .769 | .468 | .023 |
| Error (MaskerTimbre) | 66 | .384 |  |  |  |
| MaskerType×MaskerTimbre | 2 | .557 | 2.270 | .107 | .033 |
| MaskerType×MaskerTimbre×TargetTimbre | 2 | .187 | .762 | .469 | .011 |
| MaskerType×MaskerTimbre×Group | 4 | .425 | 1.732 | .147 | .050 |
| MaskerType×MaskerTimbre×TargetTimbre×Group | 4 | .234 | .955 | .435 | .028 |
| Error (MaskerType×MaskerTimbre) | 132 | .245 |  |  |  |

FIXED EFFECTS: BETWEEN SUBJECTS

| Source | df | Mean Square | F | Sig | Partial $\eta^{2}$ |
| --- | --- | --- | --- | --- | --- |
| TargetTimbre | 1 | .618 | 1.235 | .270 | .018 |
| Group | 2 | .476 | .951 | .392 | .028 |
| TargetTimbre×Group | 2 | .197 | .394 | .676 | .012 |
| Error | 66 | .501 |  |  |  |
